# Jump performance in male and female football players

**DOI:** 10.1007/s00167-019-05747-1

**Published:** 2019-10-30

**Authors:** Amelia J. H. Arundale, Joanna Kvist, Martin Hägglund, Anne Fältström

**Affiliations:** 1 Division of Physiotherapy, Department of Medical and Health Sciences, Linköping University, Linköping, Sweden; 2 Division of Physiotherapy, Department of Neurobiology, Care Sciences and Society, Karolinska Institutet, Stockholm, Sweden; 3 Football Research Group, Linköping University, Linköping, Sweden; 4 Region Jönköping County, Rehabilitation Centre, Ryhov County Hospital, 551 85 Jönköping, Sweden

**Keywords:** Sex, Soccer, Knee, ACL, Anterior cruciate ligament, Valgus, Drop vertical jump, Tuck jump, Prevention, Rehabilitation

## Abstract

**Purpose:**

To examine differences between men and women football players in clinically feasible jumping measures.

**Methods:**

Female football players (*N* = 46, ages 16–25) were matched based on age, training frequency, and playing position with 46 male players. All players performed the tuck jump and drop vertical jump (DVJ). DVJ was assessed quantitatively for valgus knee motion and probability of a high peak knee abduction moment (pKAM), as well as sagittal plane hip, knee, and ankle angles, and qualitatively with visual assessment of the player’s knees upon landing; graded as good, reduced, or poor control.

**Result:**

Women had higher total tuck jump scores (5 ± 2) (more technique flaws), than men (3 ± 2, *P* < 0.01). The quantitative analysis of the DVJ found that men had greater asymmetries between limbs, but women landed bilaterally in more knee valgus (interaction *P* = 0.04, main effect of sex *P* = 0.02). There was no difference in pKAM (interaction n.s.). Women also landed in less hip flexion (*P* = 0.01) and ankle dorsiflexion (*P* = 0.01) than men. The qualitative DVJ analysis found that more women (48%) had poor knee control compared to men (11%, *P* < 0.01).

**Conclusions:**

The results indicate that women perform worse on the tuck jump assessment than men. The results support previous findings that women land in more knee valgus than men, but also found that men may have larger asymmetries in knee valgus. These results from clinically feasible measures provide some suggestions for clinicians to consider during ACL reconstruction rehabilitation to enhance performance.

## Introduction

Differences in knee injury incidence, particularly anterior cruciate ligament (ACL) injury incidence, exist between men and women football players [33]. Women football players are at a 2–3 times higher risk for an ACL injury compared to their male counterparts [27, 35, 36], with the risk for women collegiate football players in the United States being almost four times higher [33]. Although it is unclear if there are differences in second ACL injury incidence based on sex [37], there may be differences in recovery of knee function after ACL reconstruction. One year after ACL reconstruction women have shown larger quadriceps strength asymmetries [12], as well as lower self-reported knee function than men [14].

Both men and women commonly have asymmetries in strength and functional performance after ACL reconstruction [30, 31]. For women, asymmetries or differences between legs in movement patterns, particularly knee abduction, have been implicated in second ACL injury risk [26]. However, these asymmetries may not be unique to women after ACL reconstruction. A study comparing women football players approximately 18 months after ACL reconstruction and healthy sex/age/skill level-matched controls found no differences between groups in asymmetry [6]. In fact, the control group bilaterally had more frontal plane knee motion during a drop vertical jump (DVJ) test and a higher probability of a high peak knee abduction moment (pKAM) than players who had undergone ACL reconstruction [6].

Hewett et al. [10] described four common neuromuscular deficits, more often seen in women, that potentially could contribute to ACL injury; (1) an increased knee valgus in the frontal plane during landing, (2) less flexion angle during landing using quadriceps to stabilize the knee joint, (3) asymmetrical landing, and (4) poor ability to control the trunk. The tuck jump assessment and DVJ are clinical tests commonly performed to assess high-risk movement patterns and progress during ACL reconstruction rehabilitation [19, 20]. The tuck jump is a quick clinical test that involves the player jumping continuously for 10 s [8]. The tuck jump is considered to be a more demanding test, potentially also measuring endurance, compared with DVJ, which may better reflect sport-specific jumping activities [22]. During the tuck jump players are graded on ten different technique flaws according to Herrington et al. [8], such as, e.g. landing in knee valgus, feet not shoulder width apart or knees not reaching parallel to the ground at peak jump. Jump-landing technique measured with the tuck jump improves with maturation, however, young women athletes demonstrate more knee valgus at landing and fatigue compared to men regardless of the maturation status [4, 29].

There is little information on tuck jump and DVJ asymmetries in men football players, regardless of ACL status. The most clinically feasible assessment of the DVJ is examining knee abduction via video, however the DVJ is more commonly assessed in the literature via three dimensional (3D) motion analysis. Using common, clinically feasible tests to examine sex differences, results can easily be interpreted by clinicians and may provide insight on the sex differences in injury incidence.

The purpose of this study was to examine differences between men and women football players matched regarding age, playing position and training frequency in tuck jump and drop vertical jump test. The hypothesis was that women would have higher tuck jump scores (more technique flaws) than men, and more frequently have flaws in knee valgus upon landing; as well as more asymmetry, bilaterally more knee valgus motion, a higher probability of high peak knee abduction moment, and worse knee control during the DVJ.

## Materials and methods

The women in this study have been previously reported on in a larger cohort study examining women football players after ACL reconstruction and their uninjured peers [6]. Study methodology is presented here in brief as the methods are similar to the previous study [6]. All players received written and verbal information about the study, and gave written informed consent prior to testing. The study was approved by the Regional Ethical Review Board (Dnr 2012/24-31, 2013/75-32, 2017/324-32).

**Fig. 1:**
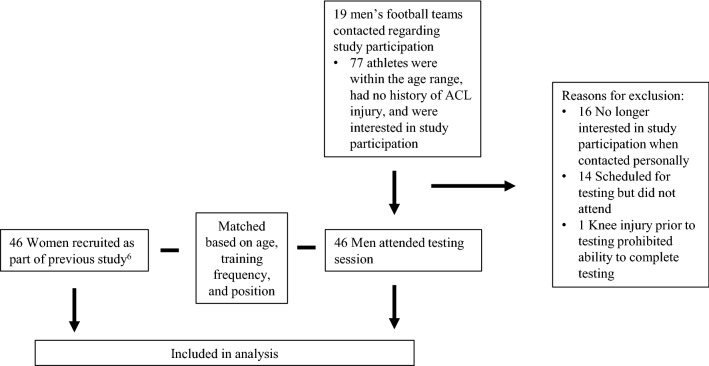
Study flowchart

Inclusion criteria for this study were football players, between the ages of 16–25, with no history of ACL injury. The forty-six women included in this study were recruited as previously described [6], and selected from the larger female cohort because appropriately matched male football players were identified. The forty-six male football players were recruited from local football teams via word of mouth, coaches, and short presentations by the researchers to the team and matched to the women based on age, training frequency, and playing position (Fig. 1).

Players performed a standardized warm-up, followed by one trial of the tuck jump and three trials of the DVJ. Both the tuck jump and DVJ were preceded by a few familiarization practice jumps. The warm-up involved running exercises typical for football players, ten squats, ten toe raises, and 1 min of jumping rope [6]. All activities were performed in the players’ own athletic shoes and clothing.

### Tuck jump

Players were instructed to stand with their feet shoulder width apart, bringing their thighs parallel to the floor, and landing in the same place. Players were instructed to immediately start the next jump upon landing and were filmed (two Panasonic HC-V500M video cameras, one in the frontal and one in the sagittal plane) as they jumped continuously for 10 s. Video was recorded at 50 Hz with advanced video coding high definition at 1080/50p. The tuck jump was analyzed according to a clinician friendly screening tool [6, 8]. The screening tool consists of ten criteria, each scored as either 0 indicating no flaw or 1 indicating flawed technique, for a total score ranging from 0 to 10. Players were classified as having an abnormal tuck jump score if their total tuck jump score was ≥ 6. This cutoff was previously proposed to indicate players who might be at a higher risk for ACL injury and might benefit from injury prevention programs. Grading was performed, by one researcher (AA), according to Herrington et al. [8] at normal video speed, reviewing each plane three times.

### Drop vertical jump

Players were given standardized instructions to drop down off the box (31 cm) and immediately jump as high as possible reaching with both hands towards a ball suspended above them. The first landing of the DVJ was assessed with two different methods, quantitatively [23] and qualitatively [25, 34]. To simplify measurements and increase visibility, the athlete’s greater trochanter, the lateral knee joint line, fibular head, lateral malleolus, patella tendon, and center of the patella were identified by palpation and marked with a marker pen. Three DVJ trials were filmed using the same one camera in the frontal and one in the sagittal plane [6].

#### DVJ quantitative analysis

As previously reported, the worst assessed jump of the three trials, summarized from all criteria, was used in the quantitative analysis [6]. The worst jump was chosen to represent the player’s potentially highest risk movement pattern, which could be overlooked if calculating the average of the three attempts. As described previously [6], each jump was given one point on the following criteria: if the feet left the box at different times, if the feet landed at the different time, if the feet were not parallel on landing, if the feet were rotated on landing, if there was knee valgus on landing, if the feet were not approximately shoulder distance apart, and if there was any weight displacement. The jump which had the most points, was deemed the worst jump and used in the analysis. In accordance with the previous study [6], the valgus knee motion was measured using Dartfish ProSuite (Dartfish Ltd, Fribourg, Switzerland) and calculated in centimeters as the medial displacement of the knee in the frontal plane from initial contact to peak knee flexion/the end of the deceleration phase of the DVJ. Valgus knee motion was inputted as zero if the athlete’s knee displacement was lateral from initial contact to peak knee flexion. A categorical variable (knee displacement) was also created based on the athlete’s frontal plane knee motion, grouping players based on if their knee displaced medially (valgus), laterally (varus), or no displacement (neutral), from initial contact to peak knee flexion. A nomogram was used to predict the probability of high knee abduction moment (pKAM) [23, 24], ranging from 0 (lowest) to 100% (highest). The nomogram is based on the player’s weight, tibia length, knee motion in the frontal plane, and knee flexion range of motion, and a surrogate value for hamstring–quadriceps ratio (multiplying the player’s mass by 0.01 and adding the resultant value to 1.10) [21, 23, 24].

The sagittal plane hip, knee, and ankle angles were measured at peak knee flexion (measurements were performed on the left leg only, as this side faced the frontal plane camera) using Dartfish ProSuite. The knee:ankle separation ratio was also calculated by dividing the distance between the center of the patella at peak knee flexion by the distance between the great toes (point estimated in Dartfish). One researcher quantitatively analyzed the women’s DVJs (IM), another analyzed the men’s (AA) and performed the qualitative analysis on all players. Inter-rater reliability testing was performed on three jumps of three women not included in this study (nine jumps total) with ICCs ranging from 0.82 to 0.99.

#### DVJ qualitative analysis

The qualitative analysis of the DVJ used a visual assessment first presented by Stensrud et al. [25, 34] Using the frontal plane view the athlete’s ability to control their knees during DVJ landing was subjectively graded on a 0–2 scale (0 = good control, 1 = reduced control, 2 = poor control). As previously described, good control was assigned when there was no obvious valgus motion of either knee, no mediolateral motion of the knee, and the knees were in line with the toes. Reduced control was indicated when there was slight mediolateral movement and/or slight valgus position of either or both knees. Poor control was assigned when players landed with knee valgus on at least one knee, alignment of the knees and toes was poor, and there was a substantial amount of mediolateral movement of the knee during landing [25]. Per the previous studies methodology [25, 34], each trial was viewed once and the trial with the highest score was used in the analysis. One researcher (AA) assessed all players, 2 months after the quantitative assessment.

### Statistical analysis

Mean ± standard deviation or absolute values with percent were calculated for descriptive statistics. One-way ANOVAs were used to compare men and women with regards to demographics and anthropometrics. Chi squared tests were used to compare the sexes with regards to limb dominance (based on preferred kicking leg), position, and training frequency.

A one-way ANOVA was used to compare men and women with regards to total tuck jump scores and Chi-squared tests were used to compare the frequency of each technique flaw during the tuck jump. A Fischer’s exact test was also used to determine if there was a difference in the number of men and women categorized as having an abnormal tuck jump score (total score ≥ 6).

#### DVJ quantitative analysis

Two-way repeated measures ANOVAs with planned least squares comparisons were used to compare the difference between limbs in men and women with regards to valgus knee motion and pKAM. Planned comparisons were the interaction effects. Fischer’s exact test was used to compare knee displacement (varus, neutral, valgus) between men and women. One-way ANOVAs were used to compare hip, knee and ankle angle at peak knee flexion as well as knee:ankle separation ratio.

#### DVJ qualitative analysis

A Fisher’s exact test was used to examine if there was a difference in the number of men and women assessed as having good, reduced, or poor control.

A sensitivity power analysis indicated that using a 2 × 2 repeated measures ANOVA with alpha set at *P * ≤ 0.05, power = 0.80, with 92 players, and effect size of *np*^2^ = 0.08 could be detected. Effect sizes were considered small (*np*^2^ = 0.01), medium (*np*^2^ = 0.06), and large (*np*^2^ = 0.14) [2].

## Results

There were no differences between sexes in age, playing position, skill level or training frequency. The men were taller and heavier than the women (Table 1).

**Table 1:** Anthropometrics and demographics of the men and women included in the study

Variable	Men (*N* = 46)	Women (*N* = 46)	*P* value
Age (years)	20.5 ± 3.0	19.9 ± 2.3	n.s.
Height (m)	180.5 ± 6.6	167.4 ± 6.7	< 0.01
Weight (kg)	75.8 ± 10.8	62.6 ± 7.6	< 0.01
Playing position			
Goalkeeper	1 (2%)	2 (5%)	n.s.
Defender	11 (24%)	13 (28%)	
Midfielder	28 (61%)	24 (52%)	
Forward	6 (13%)	7 (15%)	
Skill level			
Elite	4 (9%)	5 (11%)	n.s.
Sub-elite	28 (61%)	34 (74%)	
Recreational	14 (30%)	7 (15%)	
Training frequency (training sessions/week)			
1–2	14	13	n.s.
3–4	22	25	
≥ 5	10	8	

Age, height, and weight are presented as the mean and standard deviation. Playing level was defined as elite (top two divisions of Swedish football), sub-elite (third and fourth highest divisions), and recreational (lower divisions and youth football)

### Tuck jump

**Fig. 2:**
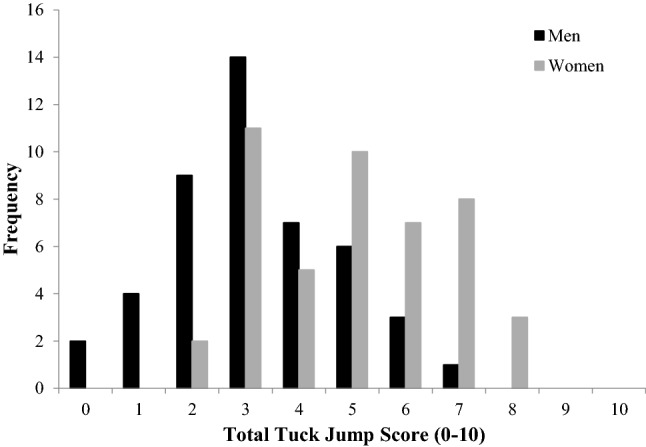
Distribution of total tuck jump scores in men and women. Total tuck jump scores range from 0 (no technique flaws) to 10 (technique flaws on all ten tuck jump items)

Women had higher tuck jump scores than men (mean ± standard deviation, 5 ± 2 vs 3 ± 2, respectively, *F*(1, 91) = 26.50, *P *< 0.01, *np*^2^ = 0.23) (Fig. 2). There was no difference between sexes in number of tuck jumps performed (women 15 ± 2 jumps, men 14 ± 3 jumps, *F*(1,91) = 2.00, n.s., *np*^2^ = 0.02). There were significantly more women with abnormal tuck jump scores (18 [39%]) than men (4 [9%], *P *< 0.01) (Fig. 2). Women more frequently had flaws in the items: thighs not parallel at peak, feet not shoulder width apart, foot placement not parallel front to back, and does not land in the same footprint (Table 2).

**Table 2:** Tuck jump technique flaws in men and women

Tuck jump assessment items	Number of players (%) scored as flawed		*P* value
	Men (*N* = 46)	Women (*N* = 46)	
Valgus on landing	15 (33%)	23 (51%)	n.s.
Thighs not equal side to side during flight	24 (52%)	25 (54%)	n.s.
Thighs not reaching parallel at peak of jump	5 (11%)	21 (46%)	< 0.01
Foot placement not shoulder width apart	22 (48%)	35 (76%)	0.01
Foot placement not parallel front to back	3 (7%)	16 (35%)	< 0.01
Foot contact timing not equal	5 (11%)	11 (24%)	n.s.
Excessive landing contact noise	17 (37%)	20 (37%)	n.s.
Pause between jumps	6 (13%)	7 (15%)	n.s.
Technique declines prior to 10 s	27 (59%)	34 (74%)	n.s.
Does not land in same footprint	23 (50%)	37 (80%)	< 0.01

### Drop vertical jump

#### DVJ quantitative analysis

There was a significant sex × limb interaction for knee valgus motion (*F*(1, 90) = 4.43, *P *= 0.04, *np*^2^ = 0.05). There was no main effect of limb (n.s., *np*^2^ = 0.04), but there was a main effect of sex (*P *= 0.02, *np*^2^ = 0.06), indicating that regardless of limb women had more knee valgus motion than men (Table 3). There was no significant sex × limb interaction for pKAM (*F*(1, 90) = 0.61, n.s., *np*^2^ = 0.01), nor main effects of sex (n.s., *np*^2^ = 0.02), or limb (n.s., *np*^2^ = 0.03) (Table 3). There was no significant difference between the number of men and women in each knee displacement category on the dominant limb (n.s.) (Table 4), however there was a difference on the non-dominant limb (*P *< 0.01).

**Table 3:** Knee valgus motion and pKAM (DVJ quantitative assessment) men and women

	Men (*N *= 46)		Women (*N* = 46)		*P* value
	Dominant	Non-dominant	Dominant	Non-dominant	
Knee valgus motion (cm)	4.1 ± 3.2	2.3 ± 2.8	4.4 ± 3.3	4.5 ± 3.4	Interaction: 0.04 Main effect of limb: n.s. Main effect of sex: 0.02
pKAM (%)	55.1 ± 41.8	64.5 ± 36.7	65.3 ± 27.3	68.4 ± 25.1	Interaction: n.s. Main effect of limb: n.s. Main effect of sex: n.s.

**Table 4:** Number of men and women in each knee displacement category (DVJ qualitative assessment)

	Varus	Neutral	Valgus	*P* value
Dominant limb knee displacement				
Men	7	3	36	n.s.
Women	2	6	38	
Non-dominant limb knee displacement				
Men	15	4	27	< 0.01
Women	3	2	41	

At peak knee flexion men had larger hip flexion (*F*(1, 91) = 6.53, *P *= 0.01, *np*^2^ = 0.07) and ankle dorsiflexion angles (*F*(1, 91) = 7.87, *P *= 0.01, *np*^2^ = 0.08) than women (Table 5). There was no difference between sexes in knee flexion angle (*F*(1, 91) = 1.85, n.s., *np*^2^ = 0.02). There was also no difference between men and women in knee:ankle separation ratio (women 1.07 ± 0.35, men 1.10 ± 0.27, *F*(1, 91) = 0.23, n.s., *np*^2^ < 0.01).

**Table 5:** Hip, knee, and ankle angles at peak knee flexion

	Men (*N* = 46)	Women (*N* = 46)	*P* value
Hip angle (°)	138.9 ± 9.3	134.0 ± 9.0	0.01
Knee angle (°)	146.3 ± 7.1	144.2 ± 6.7	n.s.
Ankle angle (°)	69.1 ± 8.8	64.2 ± 8.2	0.01

#### DVJ qualitative analysis

There was a significant difference between men and women based on the DVJ qualitative assessment. The number of players with good control was similar, but more women had poor control and more men had reduced control. There were 20 (43%) women with good, 4 (9%) with reduced, and 22 (48%) with poor control. In contrast, there were 22 (48%) men with good, 19 (41%) with reduced, and 5 (11%) with poor control (*P *< 0.01).

## Discussion

The results of this study indicate that there are differences in clinical jumping measures between men and women who play football. Women had higher tuck jump scores, indicating more technique flaws, than men. During DVJ women had more valgus knee motion bilaterally than men, however men had more asymmetry in their knee valgus. There was no difference between men and women in pKAM or knee:ankle separation ratio, however women landed in less hip flexion and ankle dorsiflexion. According to the qualitative assessment, more women had poor knee control during the DVJ landing than men. The results of this study provide some insight into sex differences in jumping performance that could be related to ACL injury risk [9], and using clinically feasible measures provide some suggestions for clinicians to consider during ACL reconstruction rehabilitation.

The tuck jump has been studied in women of various ages, sports and skill levels [4–8, 15, 22, 32], but fewer studies report results in men [4, 5, 7, 28]. The women in this study had tuck jump scores similar to those previously reported in collegiate women athletes [32]; however, the women’s scores were worse than their matched male counterparts in the present study. Significantly more women (39%) had abnormal tuck jump scores (total score ≥ 6) compared to men (9%). Prior to puberty it is thought that boys and girls have similar strength and neuromuscular control, however through/after puberty women’s strength and dynamic control over their knee joint may decrease relative to men’s [11]. The higher tuck jump scores in women seems to support that women may have worse neuromuscular control than men. Women were more likely to have flaws in thighs not reaching parallel, feet not shoulder width, feet not parallel at landing, and does not land in the same footprint. According to the factor analysis performed by Lininger et al. [15] these results could indicate that, compared to men, women have more deficits in proximal control (a lack of hip strength to control the knees and feet during landing) and more of a distal landing pattern (greater strength in the quadriceps compared to the hip extensors leading to landing with the quadriceps and hamstrings co-contracted with a flatter foot). Looking at the frequency of flaws overall, two of the three most frequent flaws seen in the men and the women were the same. These two flaws, technique declines before 10 s (flawed in 27 of 46 men and 34 of 46 women) and feet not shoulder width apart (flawed in 22 of 46 men and 35 of 46 women) could represent flaws that are ubiquitous among football players.

Although there was no statistically significant difference between men and women in the number of players who were marked as flawed on valgus upon landing during the tuck jump, there were differences in knee valgus motion during the DVJ. In the DVJ quantitative analysis, women had more knee valgus motion bilaterally than men, and more women were categorized as having valgus knee displacements. In the DVJ qualitative analysis, more women (almost half of the female cohort) had poor knee control; with poor knee control defined as knee valgus and/or mediolateral side-to-side knee motion on one or both limbs. The results of this study corroborate previous studies that women tend to land jumps in more knee valgus than men, potentially contributing to their higher risk for ACL injury [1]. Knowledge that women land in more knee valgus can help clinicians make proper landing technique a target for women during primary knee injury prevention programs as well as during rehabilitation, particularly after ACL reconstruction, potentially impacting the player’s risk for a subsequent injury.

This study is not the first to find women football players performing sport-related tasks in less hip flexion than men. Previous studies have observed elite and recreational adolescent women football players perform cutting and jumping tasks in more hip external rotation and less hip and knee flexion compared to their male counterparts [13, 38]. ACL injuries do not occur purely in the sagittal plane, however a more extended position combined with greater knee valgus could contribute to women’s higher risk for ACL injuries [16].

This study did not find any differences between men and women in probability of high peak knee abduction moment or knee:ankle separation ratio. We used clinical friendly tools such as 2D video rather than 3D motion analysis and force plates. In addition, the DVJ qualitative analysis was used to more reflect the reality assessment without using analysis system like Dartfish ProSuite (Dartfish Ltd, Fribourg, Switzerland) [3]. Where this study relied on a clinical algorithm to calculate peak knee abduction moment; motion analysis and force plates would have enabled the calculation of peak knee abduction moment directly. However, such equipment is quite expensive and not available in most clinics. 2D video and the available algorithms for estimating peak knee abduction moment are more accessible and clinically feasible [17, 23]. The Myer et al. algorithm estimates the probability of peak knee abduction moment ≥ 25.25 Nm, a threshold that may identify adolescent women athletes at higher risk for ACL injuries [9, 18, 23]. Unfortunately, as the 25.25 Nm threshold is not normalized to body weight and was established in adolescent women, it is not clear if it is valid in men. The knee:ankle separation ratio was established as an alternative by Mizner et al. The knee:ankle separation ratio was reportedly superior to frontal plane projection angle (the angle of the knee created from lines bisecting the thigh and shank drawn on a frontal plane view of the player at peak knee flexion of a DVJ), by accounting for 39% of the variance in knee abduction moment [17]. Of note, the knee:ankle separation ratio was also only validated in women. Thus, although this study did not find differences between men and women in clinically feasible surrogate measures for peak knee abduction moment, it is possible the results are due to the measures used. Future studies are needed to establish if peak knee abduction moment is an important risk factor in men, as well as identify and validate clinically feasible surrogate measures of peak knee abduction moment for all populations.

The strength of this study is the homogenous cohort of men and women football players matched regarding age, playing position and training frequency. Previous findings in tuck jump have shown that tuck jump score differ to age and is evaluated in different sports and activity levels. Men and women football players’ performances in tuck jump and DVJ have previously not been compared.

## Conclusion

This study found differences between men and women in tuck jump score and knee valgus motion during DVJ. Women had higher tuck jump scores (more technique flaws), bilaterally landed in more knee valgus, and were more likely to be graded as having poor knee control during DVJ landing. For clinicians, these findings indicate that addressing knee valgus during landing may need to be a focus for women both in prevention and potentially rehabilitation. For researchers and clinicians, this study adds further knowledge of how men and women differ in performing jumping tasks, possibly contributing more information to uncovering why women are at a higher risk for knee and ACL injuries. Further, these results also provide move evidence on the tuck jump and DVJ, 2D clinically measures which are more accessible to clinicians than 3D motion analysis.

## Abbreviations

ACL: Anterior cruciate ligament
DVJ: Drop vertical jump
pKAM: Probability of a high peak knee abduction moment
